# Dairy consumption and cardiometabolic health: outcomes of a 12-month crossover trial

**DOI:** 10.1186/1743-7075-9-19

**Published:** 2012-03-20

**Authors:** Georgina E Crichton, Peter R C Howe, Jonathan D Buckley, Alison M Coates, Karen J Murphy

**Affiliations:** 1 Nutritional Physiology Research Centre, University of South Australia, GPO Box 2471, Adelaide, South Australia 5001, Australia

**Keywords:** Dairy, Milk, Obesity, Cardiometabolic health

## Abstract

**Background:**

A growing body of research suggests that regular consumption of dairy foods may counteract obesity and other components of the metabolic syndrome. However, human intervention trials are lacking. We aimed to determine the cardiometabolic health effects of increasing the consumption of reduced fat dairy foods in adults with habitually low dairy intakes in the absence of energy restriction.

**Methods:**

An intervention trial was undertaken in 61 overweight or obese adults who were randomly assigned to a high dairy diet (HD, 4 serves of reduced fat dairy/day) or a low dairy control diet (LD, ≤1 serve/day) for 6 months then crossed over to the alternate diet for a further 6 months. A range of anthropometric and cardiometabolic parameters including body composition, metabolic rate, blood lipids, blood pressure and arterial compliance were assessed at the end of each diet phase.

**Results:**

Total energy intake was 1120 kJ/day higher during the HD phase, resulting in slight weight gain during this period. However, there were no significant differences between HD and LD in absolute measures of waist circumference, body weight, fat mass or any other cardiometabolic parameter.

**Conclusion:**

Recommended intakes of reduced fat dairy products may be incorporated into the diet of overweight adults without adversely affecting markers of cardiometabolic health.

**Trial Registration:**

The trial was registered with the Australia and New Zealand Clinical Trials Registry (ACTRN12608000538347) on 24th October, 2008.

## Background

Obesity has become a worldwide health epidemic [1]. With obesity related health costs exceeding billions of dollars in both Australia [2] and the United States [3], easily implemented interventions to slow or prevent obesity via weight loss has become a health priority.

Dairy products provide over half of the dietary intake of calcium in most parts of the Western world [4]. In addition to calcium, dairy is an important dietary source of protein, vitamin D, potassium, phosphorus and magnesium. Clinical trials show that increasing dietary calcium and dairy intake can enhance weight and fat loss and preserve lean muscle mass during energy restriction, with dairy products exerting greater effects on attenuating adiposity than calcium supplementation alone [5-7]. However, other studies have failed to find any effect of a high intake of dairy food on body weight in an energy-restricted diet [8-10]. Without energy restriction, there is little evidence for the role of high-dairy or high-calcium diets in weight or fat loss. As summarised in a thorough review by Lanou and Barnard [11], of 10 randomised controlled trials examining the effects of dairy products on body weight in adults, eight studies found no effect [12-19], and the remaining two found increases in body weight with increased dairy intake [20,21]. Only one study has reported reductions in total body fat in response to a high dairy diet, without energy restriction and in the absence of weight loss [19]. These discrepancies may be due to a number of factors including aspects of study design, such as treatment length, sample size, body weight at baseline, quantity of dairy added to the diet, as well as habitual dairy or calcium intake prior to intervention or energy restriction. Clearly, the current results are conflicting and there is not enough evidence to support a beneficial effect of a high intake of dairy food on body weight or composition.

Dairy products may have potentially advantageous effects on other metabolic risk factors via beneficial effects on blood pressure [22-24], type 2 diabetes [25,26] and the metabolic syndrome [22,27-31], all of which are cardiovascular disease (CVD) risk factors. However much of what is currently known about dairy intake and cardiometabolic health comes from cross-sectional or longitudinal studies which lack the ability to show causality. The limitations within this literature to date, summarised in a recent systematic review [32], do not allow conclusions to be drawn about the specific type and amount of dairy associated with any benefit.

Evidence from high-quality randomised controlled trials is needed to ascertain the effects of long-term consumption of reduced fat dairy food on body weight and composition, in addition to other cardiometabolic measures. The aim of this study is to monitor the effects of reduced fat dairy products on the primary outcome measure, waist circumference, in addition to other markers of cardiometabolic disease, in an at-risk population. Waist circumference was chosen as the primary outcome measure as it is a simple, reproducible measure of adiposity that people can easily understand. It is hypothesised that a high intake of reduced fat dairy food will have a beneficial effect on waist circumference and other markers of cardiometabolic health.

## Methods

### Participants

Participants were recruited via multiple strategies, including advertisements seeking people to participate in a trial examining the health benefits of dairy, placed in a local newspaper, on notice-boards around the university and in several public places (local hospital, libraries and shopping centres). During the recruitment period, a short interview segment on a current affairs program was shown on local television promoting the study and discussing the possible health benefits of dairy. Interested potential volunteers were invited to an information session and pre-study screening in which some simple health measures were taken (height, weight, blood pressure) and health and dietary questionnaires were completed to determine eligibility for inclusion in the study.

Participants were overweight or obese men and women (BMI ≥ 25 kg/m^2^), aged 18 to 75 years, who had a self-reported habitually low intake of dairy (< 2 serves/day). Low dairy consumers were selected in order to be representative of the Australian population in terms of dairy consumption, with the most recent national nutritional survey indicating that the average Australian adult consumes between 1 to 1.5 serves of dairy/day [33], where one serve equates to 250 mL of milk, 40 g of cheese or 200 g of yoghurt [34]. Similarly, overweight people were chosen as they represent the majority of the population. Recent estimates indicate that 60% of Australian adults are overweight or obese [35], and these individuals have a greater likelihood of having other components of metabolic syndrome.

An upper weight limit was set at 135 kg, as this is the maximum capacity of the dual energy X-ray absorptiometry (DEXA) used to assess body composition. Other exclusion criteria included being a current smoker, diagnosed with diabetes, CVD, liver disease, renal disease or stage 2 hypertension (> 160/100 mm Hg), pregnancy or the possibility of pregnancy within 12 months. Consumption of more than 1 g of fish oil/day, regular use of appetite suppressants, weight loss medications, or any other medication that may have influenced the study outcomes prevented inclusion. Participants were excluded if they had a known allergy or intolerance to dairy or lactose, or were considered unlikely to comply with the study protocol.

Ethical approval for the study was obtained from the University of South Australia Human Research Ethics Committee and all participants provided written informed consent prior to participating. Participants were offered AUD$200 upon completion of the study to compensate them for travel expenses incurred as a result of participation in the study.

### Study design and procedure

The 12-month dietary intervention trial was conducted at the Nutritional Physiology Research Centre, Adelaide, South Australia. Participants were randomised, with stratification by age and gender, to a high dairy diet (HD; 4 serves of reduced fat dairy/day) or a low dairy control diet (LD; 1 serve of reduced fat dairy/day) for 6 months, after which they crossed over to the alternate diet for a further 6 months. Participants were instructed to continue with their normal physical activity for the duration of the study. A crossover design was implemented to enable comparison of each condition within the same individual and ensure individual differences are controlled for, thereby reducing the sample size required to find a significant effect due to increased statistical power [36]. This design was also adopted to minimise attrition and maximise participant interest and compliance by enabling each participant to experience both diet conditions and receive complimentary dairy food. Based on waist circumference, the primary outcome measure, a total sample of 34 was estimated to give 80% power to detect an effect size of 0.5 (predicted change/SD of change) at an alpha of 0.05 [37]. This represents a detectable change in waist circumference of 1.8 cm.

### High dairy intervention

During this study arm, participants were required to consume 4 serves of reduced fat dairy/d for 6 months. Fifty-six serves of dairy were given to each participant on a fortnightly basis. Verbal and written information was given to all participants specifying serving sizes for different dairy foods. The reduced fat products provided were milk, flavoured milk, natural and flavoured yogurt, and vanilla custard. One serve of dairy equated to 250 mL (1 cup) of milk, 175-200 g (1 small tub) of yogurt, and 190 g (¾ cup) of custard. Each serve provided approximately 500 kJ, 2 g of fat (1.2 g saturated fat), 9 g of protein, 19 g of carbohydrate (18 g sugars). A selection of these foods was provided based on personal preference. All participants were instructed to *incorporate *the dairy into their diet by substituting other foods for dairy in an effort to avoid increasing their overall energy intake.

Participants were permitted to consume a small amount of dairy foods other than those provided for the study, but were asked to limit this to reduced fat varieties and to consume no more than an additional 7 serves/wk. Standard serving sizes for additional dairy were 40 g (2 slices) of cheese, 100 g of cottage cheese, 30 g (1 tablespoon) of ricotta, cream cheese or cream, 90 g (2 scoops) of ice-cream, and 11 g (1 teaspoon) of butter or margarine. These are standard serving sizes as defined by the Australian Dietary Guidelines [34]. Compliance with the HD diet was measured through the completion of weekly dairy logs, in which participants recorded all dairy consumed (type and quantity), including dairy foods consumed in addition to those provided for the study. The quantity of dairy provided was reduced for participants who regularly consumed their own preferred dairy products to ensure total intake did not exceed 28 serves/week. Participants were weighed fortnightly at dairy collection visits. If a change of weight by 2 kg or more was noticed over a fortnight participants were asked if they had changed any dietary habits other than those required for the study. If volunteers were having difficulty incorporating the dairy into their diet, they were offered an appointment with a registered nutritionist to discuss ways of incorporating dairy into their diet. For those participants who were randomised to the HD arm in the first 6 months, a follow-up phone call was made approximately 2 weeks after they crossed over to the LD treatment, to check compliance with the reduced dairy intake.

### Low dairy control

When undertaking the LD treatment participants were instructed to consume their normal diet but to consume no more than 1 serve of dairy/day. Dairy products were not provided during this phase of the study as this level of intake reflected participants typical habitual intake.

### Clinic assessments

Participants had fasting clinic assessments conducted over two consecutive mornings at baseline, 6, and 12 months. Total testing time took approximately 3.5 hours. Water only was permitted on the morning of testing and participants were instructed not to undertake any physical activity prior to testing.

### Anthropometric and body composition measures

The primary cardiometabolic outcome measure was waist circumference. Other anthropometric measures included body weight, BMI, hip circumference, total body fat, and abdominal fat.

Waist and hip circumference were measured according to the International Standards for Anthropometric Assessment [38], with the subject in a relaxed standing position with arms folded across the chest. Waist circumference was measured at the narrowest point between the lower costal border (10^th ^rib) and the most superior aspect of the iliac crest. If an obvious waist narrowing was not evident, the measurement was taken at the midpoint between the inferior costal border and the iliac crest. Hip circumference was measured around the buttocks at the level of their greatest posterior protuberance, with feet positioned together. Three measures of each were taken by the same assessor, and the average value for each calculated. When possible, the same assessor measured the same subjects at each visit. Weight was measured using electronic digital scales (Tanita Ultimate Scale, Tanita Corporation, Tokyo, Japan) to the nearest 0.2 kg. Height was measured using a wall mounted stadiometer (SECA, Hamburg, Germany), and BMI was subsequently calculated as weight(kg)/height(m^2^). Body composition measures (total body and abdominal fat, lean mass and bone mineral density) were collected by DEXA (Lunar Prodigy, Lunar Radiation Corp., Wisconsin, USA).

### Cardiometabolic measurements

Cardiometabolic outcomes included systolic and diastolic blood pressure, arterial compliance, resting metabolic rate (RMR), fasting plasma glucose, triglycerides, HDL, LDL, total cholesterol, and *hs*-CRP (high sensitivity C-reactive protein, as a measure of chronic inflammation associated with adiposity).

Blood pressure and arterial compliance were measured using the HDI/PulseWave™Cardiovascular Profiler (model CR-2000, Hypertension Diagnostics Inc™, Eagen, MN, USA). Three measures were taken and the average values calculated. RMR was measured by indirect calorimetry (TrueOne 2400 Metabolic Measurement System, PARV O Medics, Sandy, Utah USA).

At each visit, blood samples were collected by venipuncture into 9 mL EDTA tubes for plasma lipids and *hs*-CRP, and into 6 ml sodium citrate tubes for plasma glucose. Within two hours, blood samples were separated by centrifugation (Universal 32R, Hettich Zentrifugen, Germany) and stored at -80 degrees so samples could be processed in batches to reduce the effects of interassay variability. Plasma lipids (plasma total cholesterol, HDL cholesterol, triglyceride concentrations), glucose and *hs*-CRP were measured using an automated spectrophotometric analyser (Konelab, Model 20xTi; Thermo Electon, Waltham, MA, USA) with standard kits. LDL cholesterol was estimated using the Friedewald formula, a procedure which correlates highly with direct ultracentrifuge measurement [39]. The triglyceride concentration was determined as the average of the values for the two separate blood samples taken at each assessment time point.

### Dietary measurements and physical activity

A 3-day weighed food record (WFR) was completed at baseline, 6, and 12 months. Individuals were required to record all food and beverages consumed over a 3 day period (2 week days and 1 weekend day), including quantities in grams. Food and beverages were subsequently entered into a computer software program (FoodWorks Professional Edition 2009 using food composition data from AUSNUT 2007 and NUTTAB 2006) for detailed dietary analyses to provide average daily nutrient intakes. A food frequency questionnaire (FFQ) was administered at baseline to gain an accurate indicator of dairy consumption over the previous 12 months prior to entry into the trial. The FFQ requests information relating to food choices, frequency, portion size, quantity and consumption rate of different food and beverage items [40]. The FFQ is a validated and reliable measure of dietary intake for use in epidemiological studies within the Australian population [41-43].

A 3-day physical activity diary (PAD) [44] was completed over the same 3 days as the WFR. For each 15 minute period subjects estimate the level of physical activity they were engaging in. Values were based on the dominant activity for that period, ranging from 1) sleeping, to 9) intense manual work or high intensity sport. The estimated energy cost in kcal/kg/15 min for each of the nine activity levels is used to calculate daily energy expenditure. This tool has been validated for use in adult populations [44].

### Data analysis

One-way ANOVA and Chi square were used to compare baseline demographic, cardiometabolic and dietary characteristics between those who completed the 12 month study (completers, n = 36), and those who dropped out of the study (n = 25). Two-sample t-tests were performed to determine whether there were any significant differences in any of the cardiometabolic or dietary factors at baseline between those randomised to HD in the first phase and those randomised to LD. Carry-over, treatment and period effects were tested according to Jones and Kenward [36]. To test for any carry-over effects, two-sample t-tests were performed to compare the sum of the cardiometabolic outcome variables (body composition, blood pressure and arterial compliance, RMR and blood measures), at the end of each period in the two groups of participants (those who started in HD and those who started in LD). There were no significant differences in the sum of outcome scores between the two groups. To test for any period effects, two-sample t-tests were performed to compare differences in cardiometabolic outcomes between the two diet periods (end of HD - end of LD) in the two groups. There were no significant differences in the change score for the two groups, indicating no period effects. We tested for any seasonal effects, to determine whether results differed for those who undertook HD in summer or winter. Using two-sample t-tests, there were no significant differences in scores for those who undertook HD in summer versus those who undertook HD in winter. To test for treatment effects, the difference in outcome scores between the first and second periods were calculated for the two groups, and these values were compared using two-sample t-tests. These results were confirmed with paired t-tests to compare the within-individual measurements at the end of each dietary phase. Paired t-tests were also used to compare the mean change of each cardiometabolic factor within each dietary phase. This takes into account the starting point for each person for each period.

Paired t-tests were used to determine whether there were any changes in diet (total energy, macronutrient intakes) between the two diet phases. Linear regression analyses were performed to determine whether any changes in dietary variables between the two diet phases correlated with any changes in cardiometabolic outcomes. All statistical analyses were conducted using SPSS for Windows, version 18.0 (SPSS Inc., Chicago, IL, USA). Data are presented as means ± standard error of the mean (SEM) unless otherwise stated. *P *< 0.05 was considered significant for the primary outcome measured. Bonferroni adjustment was applied to the level of significance for secondary outcomes to reduce the risk of Type I error due to multiple comparisons.

## Results

### Participants

**Figure 1:**
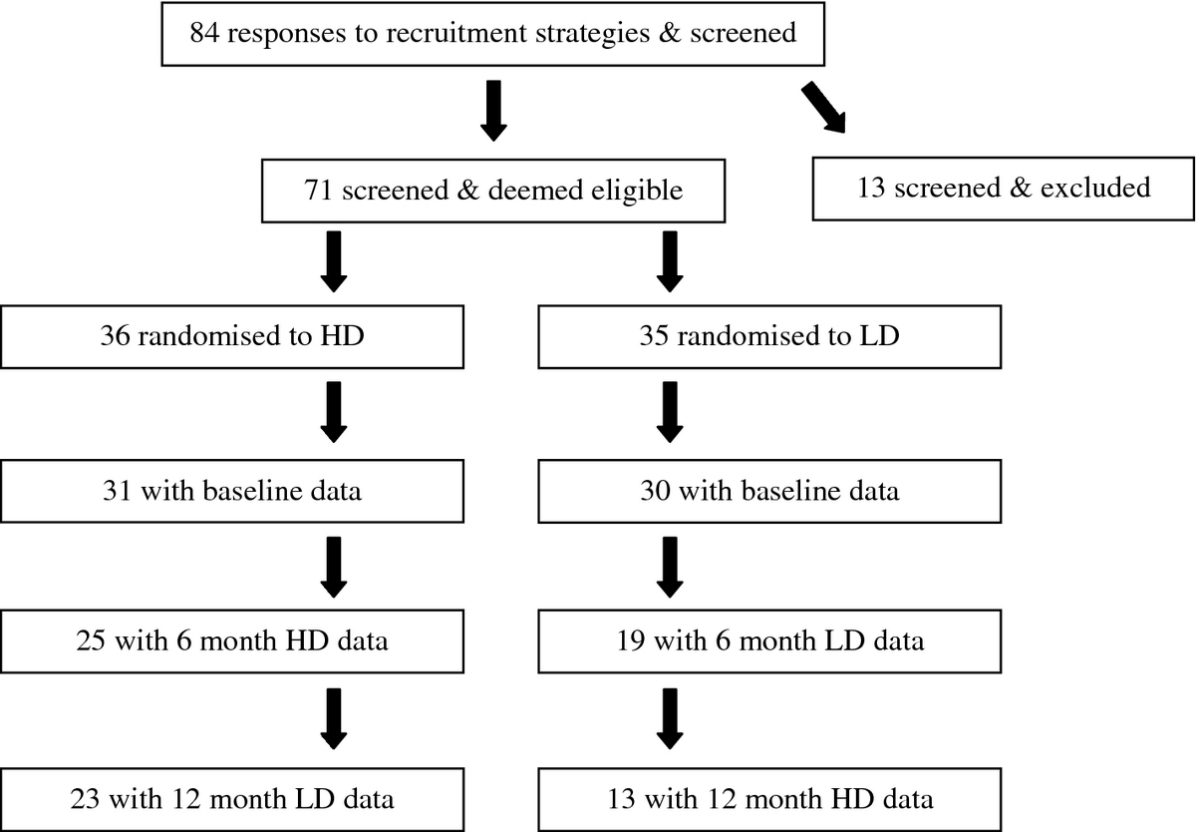
**Participant flow and attrition**. Participant flow through the study, showing study design with participant progression and attrition following initial randomisation to the HD and LD diets. HD, high dairy; LD, low dairy.

Of 84 people who were initially screened, 71 were eligible to participate, gave consent and were enrolled and allocated to diets. Sixty-one participants (18 male, 43 female), completed a baseline assessment. A further 25 participants withdrew during the course of the study. Due to the disproportionate number of drop-outs from each diet (17 initially randomised to LD and 8 randomised to HD) the numbers remaining on each diet at the end of the study were unequal. In total, 36 participants (23 initially allocated to HD and 13 allocated to LD), aged 18 to 71 years, completed the 12-month study. A summary of randomisation and participant flow is shown in Figure 1. The overall rate of attrition was 49.3%. At baseline, there were no significant differences (data not shown) in sex distribution, age, total energy or macronutrient intakes, physical activity levels, or cardiometabolic characteristics between those who withdrew during the study period (n = 25) and those who completed the study (n = 36).

The most frequent reasons cited for leaving the study were due to changes that the participant intended to make that would interfere with study outcomes, such as starting a new medication or changing diet or exercise habits. Other explanations for drop-out given were for personal reasons or because they were moving away from the study location, time commitment, or an inability to consume 4 serves of dairy/day while in the HD phase. Four participants in the LD group withdrew prior to, or at the time of crossover as they were unwilling to increase their dairy consumption.

There were no statistically significant differences in any of the cardiometabolic or dietary factors at baseline between those randomised to HD in the first phase and those randomised to LD. There was no evidence of any period, order or seasonal effects. The change between the two dietary phases in the primary outcome measure, waist circumference, in addition to secondary outcomes including body composition, blood pressure, blood lipids and glucose, arterial stiffness and RMR, did not differ significantly between those who consumed HD in the first phase and those who consumed HD in the second phase. Total energy intake and physical activity at the end of the two diet phases did not differ according to order of intervention.

### Dietary intake

At baseline, the mean intake of dairy (sum of milk, cheese, yogurt, ice-cream, and butter/margarine) was 1.9 ± 0.9 serves/day. During the HD phase, the intake of dairy foods was 28.1 ± 2.6 serves/week, equating to 4 serves/day (comprising 25.2 ± 3.1 serves of supplied dairy and 2.9 ± 2.4 serves of own dairy/week). The mean daily intakes of dairy products in the HD phase were 535 mL of plain milk, 428 mL of flavoured milk, 257 g of yogurt, and 81 g of custard.

Energy intake differed between diets (intakes taken from 3-day WFR's shown in Table 1). Mean energy intake was significantly higher (1120 ± 360 kJ/day; *p *= 0.004) during the HD phase, than during the LD control phase. The increased dairy intake during the HD phase was associated with a significantly higher intake of protein (*p *< 0.001) and carbohydrate (*p *= 0.03). The intakes of macronutrients as a proportion of total energy in the diet also different significantly between the two phases, with a higher proportion of total energy from protein (*p *= 0.011), and a lower proportion from fat (*p *= 0.006) during HD. Total sugar intake was significantly higher during HD compared with LD (*p *< 0.001). Calcium intake was significantly higher (*p *< 0.001) during HD (1452 mg/day) compared with LD (723 mg/day).

**Table 1:** Average daily dietary intakes at end of LD and at end of HD, from 3-day weighed food records

Daily dietary intakes	Baseline	End HD	End LD	Change HD-LD	*P*-value*
n	30	32	32	32	

Total energy (MJ)	8.71 ± 0.61	8.84 ± 0.41	7.73 ± 0.37	1.12 ± 0.36	< 0.01

Protein (g)	97 ± 6	110 ± 5	84 ± 3	26 ± 5	< 0.001

Carbohydrate (g)	226 ± 17	232 ± 12	209 ± 13	24 ± 10	0.03

Fat (g)	78 ± 7	69 ± 5	65 ± 4	4 ± 5	0.42

SFA (g)	31 ± 4	27 ± 2	23 ± 2	3 ± 2	0.11

PUFA (g)	11 ± 1.0	9 ± 0.9	10 ± 0.9	-1.0 ± 1.0	0.32

MUFA (g)	28 ± 3	23 ± 2	22 ± 1	1.1 ± 1.9	0.56

Alcohol (g)	7 ± 2	10 ± 2	8 ± 2	2.4 ± 1.7	0.16

Sugars (g)	110 ± 10	127 ± 8	92 ± 7	36 ± 5	< 0.001

Fibre (g)	24 ± 2	21 ± 2	22 ± 2	-0.8 ± 1.2	0.50

Calcium (mg)	1112 ± 146	1452 ± 68	723 ± 35	730 ± 74	< 0.001

Protein (% of total energy)	19 ± 0.8	22 ± 0.9	19 ± 0.7	2.6 ± 1.0	0.01

Carbohydrate (% of total energy)	40 ± 1.4	40 ± 1.2	39 ± 1.2	0.9 ± 1.4	0.52

Fat (% of total energy)	33 ± 1.1	28 ± 1.0	31 ± 0.9	-3.0 ± 1.0	< 0.01

Sugars (% of total energy)	23 ± 1.3	25 ± 1.1	20 ± 0.9	4.9 ± 0.8	< 0.001

Values are means ± SEM. HD, high dairy; LD, low dairy; MUFA, monounsaturated fatty aid; PUFA, polyunsaturated fatty acid; SFA, saturated fatty acid. *Probability of differences between the end of the 2 diet periods (paired sample t-tests)

### Anthropometric and body composition measures

There was no significant difference in the primary cardiometabolic outcome, waist circumference, between the HD and LD phases of the study (Table 2). Nor were there any significant differences in body weight, hip circumference, BMI, total body fat or abdominal fat between the two diet conditions. However, the mean changes in body weight, BMI, and hip circumference within the HD phase (change over 6 month period) were greater than within the LD phase (Table 3).

**Table 2:** Cardiometabolic variables at baseline, at end of LD and at end of HD

Variable	Baseline	End HD	End LD	Change HD-LD	*P*-value*
n	36	36	36	36	

RMR (MJ/d)	6.7 ± 0.3	6.9 ± 0.3	6.9 ± 0.3	0.06 ± 0.3	0.86

Energy expenditure (MJ/d)	16.1 ± 0.7	16.1 ± 0.7	15.9 ± 0.7	0.3 ± 0.4	0.51

Body weight (kg)	88.6 ± 2.9	90.8 ± 3.0	90.3 ± 3.0	0.5 ± 0.5	0.29

BMI (kg/m^2^)	31.5 ± 0.9	32.2 ± 1.0	32.0 ± 0.9	0.2 ± 0.2	0.20

Waist circumference (cm)	98.2 ± 2.4	98.9 ± 2.4	98.3 ± 2.5	0.6 ± 0.6	0.33

Hip circumference (cm)	114.3 ± 2.0	115.8 ± 2.1	115.4 ± 2.1	0.5 ± 0.3	0.18

Body fat (%)	43.0 ± 1.5	43.6 ± 1.5	43.5 ± 1.5	0.1 ± 0.3	0.67

Fat mass (kg)	36.6 ± 1.9	38.0 ± 2.0	37.7 ± 2.0	0.3 ± 0.4	0.46

Lean mass (kg)	48.0 ± 1.9	48.5 ± 1.9	48.3 ± 1.9	0.1 ± 0.3	0.60

Abdominal fat (kg)	2.6 ± 0.2	2.7 ± 0.2	2.7 ± 0.2	-0.01 ± 0.03	0.71

Bone mineral density (g/cm^2^)	1.3 ± 0.02	1.3 ± 0.02	1.2 ± 0.02	0.002 ± 0.004	0.65

SBP (mm Hg)	125.9 ± 2.2	131.8 ± 2.6	130.9 ± 2.3	0.9 ± 1.4	0.51

DBP (mm Hg)	69.2 ± 1.4	70.3 ± 1.5	71.3 ± 1.3	-1.0 ± 1.1	0.34

LAEI (ml/mm Hg × 100)	16.0 ± 4.7	14.9 ± 4.4	15.4 ± 5.4	-1.0 ± 0.8	0.25

SAEI (ml/mm Hg × 100)	7.0 ± 0.7	6.3 ± 0.6	7.3 ± 0.8	-1.0 ± 0.5	0.03

Plasma glucose (mmol/L)	5.8 ± 0.1	5.9 ± 0.1	5.8 ± 0.1	0.04 ± 0.5	0.64

Triglycerides (mmol/L)	1.1 ± 0.1	1.3 ± 0.1	1.2 ± 0.1	0.1 ± 0.3	0.21

Total cholesterol (mmol/L)	5.1 ± 0.2	5.4 ± 0.2	5.4 ± 0.2	-0.01 ± 0.5	0.86

HDL cholesterol (mmol/L)	1.4 ± 0.1	1.4 ± 0.1	1.4 ± 0.1	-0.02 ± 0.1	0.51

LDL cholesterol (mmol/L)	3.7 ± 0.1	3.9 ± 0.1	3.9 ± 0.1	0.002 ± 0.4	0.98

*hs*-CRP (mg/L)	6.4 ± 1.7	4.4 ± 0.8	4.9 ± 1.4	-0.5 ± 5.1	0.57

Values are means ± SEM. BMI, body mass index; DBP, diastolic blood pressure; HD, high dairy; HDL, high-density lipoprotein; *hs*-CRP, high sensitivity C-reactive protein; LAEI, large arterial elasticity index; LD, low dairy; LDL, low-density lipoprotein; RMR, resting metabolic rate; SAEI, small arterial elasticity index; SBP, systolic blood pressure. *Probability of differences between the end of the 2 diet periods (paired sample t-tests)

**Table 3:** Mean change in cardiometabolic variables during HD and LD

Variable	Mean change during HD	Mean change during LD	*P*-value*
n	36	36	

Body weight (kg)	1.8 ± 0.4	0.2 ± 0.5	0.01

BMI (kg/m^2^)	0.6 ± 0.1	0.0003 ± 0.2	0.01

Waist circumference (cm)	0.8 ± 0.6	-0.8 ± 0.6	0.14

Hip circumference (cm)	1.5 ± 0.4	0.005 ± 0.4	0.03

Body fat (%)	0.7 ± 0.3	0.1 ± 0.3	0.28

Fat mass (kg)	1.3 ± 0.4	0.2 ± 0.3	0.05

Lean mass (kg)	0.3 ± 0.3	0.01 ± 0.3	0.60

Abdominal fat (kg)	0.09 ± 0.03	0.07 ± 0.03	0.81

Bone mineral density (g/cm^2^)	-0.004 ± 0.003	-0.004 ± 0.004	0.96

SBP (mm Hg)	4.3 ± 1.6	0.8 ± 1.5	0.19

DBP (mm Hg)	0.6 ± 0.8	2.0 ± 1.0	0.40

LAEI (ml/mm Hg × 100)	-0.6 ± 0.4	0.5 ± 0.9	0.33

SAEI (ml/mm Hg × 100)	-0.9 ± 0.4	0.6 ± 0.6	0.07

Plasma glucose (mmol/L)	-0.008 ± 0.09	-0.03 ± 0.09	0.87

Triglycerides (mmol/L)	0.2 ± 0.07	-0.03 ± 0.06	0.09

Total cholesterol (mmol/L)	0.08 ± 0.07	0.2 ± 0.1	0.39

HDL cholesterol (mmol/L)	-0.01 ± 0.03	0.03 ± 0.03	0.31

LDL cholesterol (mmol/L)	0.09 ± 0.05	0.2 ± 0.1	0.53

*hs*-CRP (mg/L)	-1.2 ± 1.4	-0.09 ± 0.9	0.54

Values are means ± SEM. BMI, body mass index; DBP, diastolic blood pressure; HD, high dairy; HDL, high-density lipoprotein; *hs*-CRP, high sensitivity C-reactive protein; LAEI, large arterial elasticity index; LD, low dairy; LDL, low-density lipoprotein; SAEI, small arterial elasticity index; SBP, systolic blood pressure. *Probability of differences between the 2 means (paired sample t-tests)

### Cardiometabolic outcomes

There were no significant changes in RMR or total energy expenditure, systolic and diastolic blood pressure, fasting blood glucose, total, HDL or LDL cholesterol, triglycerides or *hs*-CRP. There was a slight decrease (1.0 ± 0.5 ml/mmHg × 100; *p *= 0.032) in small arterial elasticity index (SAEI) at the end of HD (Table 2), but this was not statistically significant after Bonferroni adjustment. Similarly, there were no differences in the amount of change within each period for any of these variables.

## Discussion

This relatively long-term crossover intervention study examined effects of increasing the intake of reduced fat dairy food in habitually low dairy consumers, without restricting overall energy intake. Participants increased their energy intake whilst consuming more dairy food, yet a comparison of absolute values at the end of each period showed no significant difference in body weight, body fat, or abdominal adiposity. The difference in energy intake of approximately 1120 kJ/day between LD and HD over 6 months equates to a difference of > 30 000 kJ/month, which would be expected to contribute to a weight gain of ~1 kg/month or a 6 kg difference in body weight over the 6 month HD diet period compared with the LD control diet. We observed a much smaller weight increase within the HD phase (mean increase of 1.8 ± 0.4 kg) than what may have been expected considering the large increase in energy intake. There was no evidence of an increase in either RMR or physical activity during the HD phase which might have balanced the increased energy intake. However faecal excretion of fat was not assessed and the increased calcium intake during HD might have promoted the formation of calcium-soaps in the intestine resulting in increased fat excretion and reduced fat absorption, thus accounting for only the small weight increase observed despite a higher energy intake during HD.

The finding of minimal weight gain despite a higher energy intake with HD is similar to that of Zemel et al. [45] who compared a high vs. low intake of dairy intake on weight maintenance following a period of weight loss. Participants were randomised to either recommended dairy (> 3 servings/day) or low dairy (< 1 serving/day) for a 6 month maintenance phase. The recommended dairy group had a significantly higher energy intake compared to the low dairy group, but exhibited no gain in body weight, waist circumference, total body fat, or abdominal fat.

Two other studies have shown beneficial effects from high dairy and calcium intakes on anthropometric measures without energy restriction. Zemel et al. [19] demonstrated that increasing the dairy intake in obese African Americans to 3 servings/day in the absence of energy restriction for 24 weeks had beneficial effects on body composition, reducing total body and abdominal fat and increasing lean mass, while maintaining a stable body weight. In a 2-year exercise intervention study, Lin et al. [46] showed that high intakes of dairy calcium (but not non-dairy calcium), adjusted for energy intake, were associated with weight loss and body fat loss, independent of exercise. They also noted an interesting interaction between calcium and energy intake, whereby in those with lower energy intakes, total or dairy calcium, but not energy intake, negatively predicted weight and fat mass. In those with energy intakes greater than the mean (~7850 kJ), energy intake, but not calcium intake positively impacted on body weight and body fat. There was no difference in calcium intake between the low and high energy intake groups.

Furthermore, two studies [9,10] have reported similar weight and fat loss in high-dairy calcium groups in combination with energy restriction (500 kcal/day deficit), compared with low or moderate-dairy calcium groups for 12 months, despite a higher energy intake (~150 kcal/day) in the high dairy groups. Together, these data suggest that high dairy consumers appear to be able to consume more food without adversely affecting body composition.

It is currently thought that dairy foods may alter overall energy balance by increasing fat oxidation and by decreasing the absorption of fatty acids. A number of studies have shown that increased dietary calcium intake, including calcium from dairy [47-50], increases fat oxidation. The effects of calcium on fat mass are proposed to occur via the regulation of the calcitrophic hormones, parathyroid hormone (PTH) and 1,25-dihydroxyvitamine D (1,25(OH)_2_D). These hormones are produced in response to low-calcium diets, stimulating the influx of intracellular calcium in adipocytes, promoting lipogenesis and increased adiposity [51,52]. Increasing dietary calcium can suppresses circulating PTH and 1,25(OH)_2_D, with a corresponding increase in lipolysis and fat oxidation [47,51,52]. Findings to date suggest the impact of calcium on adiposity may be dependent upon energy intake, and likely enhanced in a state of negative energy balance. Further exploring this possibility, Melanson et al. [48] found that a high dairy and calcium diet for 1 week increased fat oxidation under conditions of acute energy deficit, but not when energy was maintained. In contrast, in the absence of energy deficit Gunther et al. [47] observed a significant increase in whole-body fat oxidation after 1 year of increased dairy product consumption, compared with a low dairy group control, yet no weight change was observed, despite a slightly higher energy intake in the dairy intervention group compared with control [47], as was the case in the present study.

The small increase in body weight despite the higher energy intake during HD in the present study does not appear to be due to an increase in fat oxidation. Energy expenditure estimated from 3-day physical activity diaries was slightly higher at the end of the HD phase than at the end of LD, but this was not statistically significant. There was no difference in RMR between the two diet phases, although it is possible that the assessment of RMR using indirect calorimetry may not have been sensitive enough to detect a small change in metabolic rate. Those studies that have reported increased fat oxidation in response to increased dairy or calcium intakes have typically used whole-room indirect calorimetry [48,49], enabling measurement of energy expenditure and substrate oxidation over 24 hours during different levels of activity (i.e. during sleep and active periods), or have used the doubly labeled water technique [50]. Thus, while our study did not demonstrate an increase in fat oxidation with HD, it is possible that our method of assessing this outcome was not sensitive enough to detect any small change.

It is more feasible that the increase in dairy calcium during the HD phase reduced fat absorption, thereby preventing more substantial gains in body weight or fat mass. Calcium intake during the HD phase, 1452 mg/day, is very similar to the calcium intake of 1325 mg/day for the dairy group (approximately 3 serves of dairy/day) during weight maintenance in the Zemel et al. [45] study where similarly, despite a higher energy intake compared to the low dairy group, the dairy group exhibited no gain in body weight, waist circumference, total body fat, or abdominal fat. The calcium intake during HD is also comparable to a study conducted over a similar time frame (24 weeks) and without energy restriction, which showed a decrease in total body fat and trunk fat, and increase in lean mass following a HD diet (3 serves of dairy, 1200 mg/day of calcium) [19].

Several human studies have demonstrated that dietary calcium increases faecal fat excretion by forming insoluble soaps with fatty acids in the intestine, decreasing fatty acid absorption, resulting in energy and weight loss [53-55]. Jacobsen et al. [53] showed that a short-term increase in calcium intake (1800 mg/day) from reduced-fat dairy products, in conjunction with normal protein intake (15% of total energy), increased faecal fat and energy loss by approximately 312 kJ/day, which corresponds to a weight loss of 3.5 kg/year. Interestingly, there was no effect on energy loss when protein intake (23% of total energy) was high. Jacobsen et al. [53] suggest that in the presence of high dietary protein, more calcium may be absorbed, and secondly may bind to protein, making less available to bind with fatty acids. In our study, calcium intakes were a little lower, but 22% of energy was derived from protein at the end of the HD phase, which may have confounded the ability of calcium to exert even greater effects.

We cannot be absolutely certain that energy intake was greater whilst participants were assigned to HD, even though our results indicate this was the case. The lack of contact with investigators while in the LD phase may have led participants to under-report intakes during this phase. As they were not visiting the centre fortnightly and receiving the same attention as the HD group, they may have been less likely to pay close attention to what they were eating, thus introducing selective bias.

The increased consumption of dairy foods did not affect other cardiometabolic parameters including blood pressure, which is postulated to benefit from dairy consumption [24,56-58]. However, blood pressure reductions are usually demonstrable only in hypertensives and our population was clearly normotensive. A 2.9 mmHg reduction in systolic blood pressure was observed in a recent study of similar design, whereby overweight participants consumed 3 serves of low-fat dairy/day for 8 weeks and then crossed-over to a carbohydrate-rich control period for 8 weeks [58]. However, the baseline blood pressure was considerably higher for these participants (mean systolic blood pressure 135 mmHg, diastolic blood pressure 88 mmHg than in the present study (systolic blood pressure 126 mmHg, diastolic blood pressure 69 mmHg).

There was a slight reduction in SAEI in this study at the end of HD compared with the LD control period. However, this cannot be regarded as significant due to the possibility of a type I error resulting from multiple comparisons. It was encouraging to observe that a high consumption of reduced fat dairy foods did not have an adverse effect on blood lipids, blood glucose or *hs*-CRP. There was no significant change in bone mineral density; however, a longer time frame may be required to detect this. The shorter-duration study by van Meijl and Mensink [58] similarly did not find any change in other cardiometabolic risk parameters, including serum lipids, plasma glucose, insulin, CRP, or body weight between the two diet conditions, although they found that low-fat dairy consumption had a beneficial effect on the action of the inflammatory marker TNF-α [59]. The lack of any beneficial effect of HD vs. LD is also consistent with Wennersberg et al. [60], who examined dairy intake in relation to components of the metabolic syndrome in overweight adults. In this parallel-group intervention trial, overweight adults were similarly assigned to either a milk diet (3-5 portions/day) or control (habitual) diet for 6 months and no significant differences were found in body weight, body composition or other components of the metabolic syndrome. Perhaps the time frame in the present study and that of Wennersberg et al. [60] may have been too short to observe any improvement in cardiometabolic parameters.

Some limitations to the present study must be acknowledged. The type of dairy foods provided to participants may have influenced their ability to incorporate the dairy into their diet and subsequent energy balance. The increase in energy intake during the HD phase and subsequent small gain in body weight reflects an inability to incorporate the dairy food into the diet. Participants were predominantly provided with milk, yogurt and custard, all rich in whey protein. It may have been easier for participants to incorporate cheese products (e.g., cream cheese, cheddar cheese, ricotta) into their diet without an overall increase in energy intake.

Additionally, a larger sample size would have been more desirable. Sustaining the involvement of individuals and compliance with the dietary requirements throughout the lengthy intervention period and data collection process presented a major challenge. The difficulties in running long term dietary intervention trials based on our experiences in the present study and the biases incurred as a result of high drop-out, including the general applicability of results and effect on power, are summarised in a separate manuscript. A larger sample in the current study would have permitted us to detect smaller changes in cardiometabolic measures that may be more likely in dietary intervention studies without energy restriction. Another acknowledged limitation is the lack of a wash-out period. Our simple design (without a wash-out and re-assessment of baseline) is unable to accurately determine whether there were any carry-over effects. However, the exceptionally long intervention period (6 months) was expected to enable complete habituation to the alternate diet.

Despite these challenges, there are a number of strengths to the present study. This is one of the first intervention trials examining dairy intake without energy restriction, in relation to cardiometabolic health. Adherence to consuming 4 serves of dairy food/day during the HD phase was excellent, and we were therefore able to increase the dairy consumption of low consumers to meet the current Australian Dietary Guidelines recommendation for 2 to 3 serves of milk, yogurt or cheese each day for women and 2 to 4 serves for men [34,61]. Consequently recommended intakes of calcium, 31% higher at the end of HD compared with baseline, were also met. Of the 37 participants who completed the HD intervention and interview, 27 reported that at the end of the study they would be more likely to consume more dairy than they did prior to the study. Although the accuracy of actual dietary intake from self-reported food records may be poor [62], a strength of the study is that participants served as their own control, so that they presumably would have over- or under-reported intakes to a similar extent at each time point.

## Conclusions

Few studies have assessed the effects of dairy foods on measures of body weight and other cardiometabolic parameters without restricting energy intake. The results from our clinical trial are consistent with an anti-obesity effect of dairy foods, suggesting that recommended intakes of reduced fat dairy products may be incorporated into the diet without weight or fat gain. If overall energy intake can be carefully controlled, reduced fat dairy products may be utilised for preventing and managing overweight and obesity.

## Abbreviations

1,25(OH)_2_D: 1,25-dihydroxyvitamine D; CVD: Cardiovascular disease; DEXA: Dual energy X-ray absorptiometry; FFQ: Food frequency questionnaire; HD: High dairy; HDL: High-density lipoprotein; *hs*-CRP: high sensitivity C-reactive protein; LAEI: Large arterial elasticity index; LD: Low dairy; LDL: Low-density lipoprotein; PTH: Parathyroid hormone; RMR: Resting metabolic rate; SAEI: Small arterial elasticity index; WFR: Weighed food record

## Competing interests

The authors declare that they have no competing interests.

## Authors' contributions

PRCH, JDB and AMC designed the research, reviewed and contributed to the manuscript. KJM designed the research, collected the data, reviewed and contributed to the manuscript. GEC collected and analysed the data, and wrote the first draft of the manuscript. All authors read and approved the final manuscript.

## Funding

This research was supported by the South Australian Government as part of its 10 Year Vision for Science, Technology and Innovation (STI10).
